# Localization of DIR1 at the tissue, cellular and subcellular levels during Systemic Acquired Resistance in *Arabidopsis *using DIR1:GUS and DIR1:EGFP reporters

**DOI:** 10.1186/1471-2229-11-125

**Published:** 2011-09-06

**Authors:** Marc J Champigny, Heather Shearer, Asif Mohammad, Karen Haines, Melody Neumann, Roger Thilmony, Sheng Yang He, Pierre Fobert, Nancy Dengler, Robin K Cameron

**Affiliations:** 1 Department of Biology, McMaster University, Hamilton, ON L8S 4K1 Canada; 2 Department of Cell and Systems Biology, University of Toronto, 25 Willcocks Street, Toronto, ON, M5S 3B2, Canada; 3 Department of Plant Biology, Michigan State University, East Lansing MI, 48824 USA; 4 Plant Biotechnology Institute, 110 Gymnasium Place, Saskatoon, SK S7N 0W9 Canada; 5 USDA-ARS, Western Regional Research Center, Crop Improvement and Utilization Research Unit, 800 Buchanan St., Albany, CA, 94710 USA

## Abstract

**Background:**

Systemic Acquired Resistance (SAR) is an induced resistance response to pathogens, characterized by the translocation of a long-distance signal from induced leaves to distant tissues to prime them for increased resistance to future infection. DEFECTIVE in INDUCED RESISTANCE 1 (DIR1) has been hypothesized to chaperone a small signaling molecule to distant tissues during SAR in *Arabidopsis*.

**Results:**

DIR1 promoter:DIR1-GUS/*dir1-1 *lines were constructed to examine DIR1 expression. DIR1 is expressed in seedlings, flowers and ubiquitously in untreated or mock-inoculated mature leaf cells, including phloem sieve elements and companion cells. Inoculation of leaves with SAR-inducing avirulent or virulent *Pseudomonas syringae *pv *tomato *(*Pst*) resulted in Type III Secretion System-dependent suppression of DIR1 expression in leaf cells. Transient expression of fluorescent fusion proteins in tobacco and intercellular washing fluid experiments indicated that DIR1's ER signal sequence targets it for secretion to the cell wall. However, DIR1 expressed without a signal sequence rescued the *dir1-1 *SAR defect, suggesting that a cytosolic pool of DIR1 is important for the SAR response.

**Conclusions:**

Although expression of DIR1 decreases during SAR induction, the protein localizes to all living cell types of the vasculature, including companion cells and sieve elements, and therefore DIR1 is well situated to participate in long-distance signaling during SAR.

## Background

Acquired resistance, or "immunization" of plants was originally documented more than seventy years ago in a review published by Kenneth Chester in which varying degrees of immunity were observed in plants that had recovered from an initial pathogen attack [1]. The term systemic acquired resistance (SAR) was originally used by Ross to describe systemic resistance induced by necrosis-causing viruses in tobacco [2] and is more generally defined as a defense mechanism induced by a localized infection that results in broad-spectrum resistance in distant tissues to normally virulent pathogens [3,4].

Research using tobacco, cucumber and, more recently, *Arabidopsis *models indicates that SAR occurs in distinct stages. The first, or induction, stage is initiated when a necrosis-causing pathogen infects a leaf and results in either the formation of a localized hypersensitive response (HR) and local resistance, or in disease-induced necrosis [3]. A recent report demonstrated systemic immunity in the absence of necrotic cell death in the induced leaf [5], highlighting the fact that the precise cellular mechanisms governing the initiation of SAR are still unclear. Formation of the necrotic lesion results in a 10 to 50-fold accumulation above basal levels of the plant defense hormone, salicylic acid (SA),[6-11] and in the expression of pathogenesis-related (*PR*) genes [6,11,12]

During the initiation stage of SAR, a mobile signal or signals is induced to travel and is later perceived in distant, uninfected tissues. Several lines of evidence indicate that the signal travels through the phloem, including girdling experiments in tobacco that reduce the translocation of molecules through phloem tissue. Additionally, the pattern of sucrose transport from source to sink leaves in *Arabidopsis *was similar to transport of the SAR signal from induced leaves to protect upper leaves against *Pseudomonas syringae *pv *maculicola *(*Psm*). Although these and other experiments [reviewed in 13] suggest the SAR signal is phloem-mobile, cell-to-cell movement down the petiole, or a combination of these two modes of transport cannot be ruled out.

The discovery that SA levels in the phloem rise dramatically in SAR-induced tobacco [9] and cucumber [10] led to the hypothesis that SA itself may be a SAR mobile signal [14]. SA was shown to be critically involved in the SAR pathway because transgenic tobacco plants expressing a salicylate hydroxylase gene (*NahG*) were unable to accumulate SA or to manifest a SAR response [14]. However, a number of experiments provide evidence that SA is not a SAR mobile signal. Cucumber plants in which induced leaves were detached prior to the accumulation of SA in their petioles still manifested a SAR response in systemic tissue [15]. Furthermore, grafting experiments utilizing transgenic *NahG *tobacco demonstrated that *NahG*-expressing rootstocks blocked in the accumulation of SA were nonetheless competent to translocate a mobile signal to the scion [16].

The establishment phase of SAR involves the perception of the mobile signal(s) in distant tissue, resulting in a modest accumulation of SA and expression of *PR *genes in *Arabidopsis *and tobacco [7,8,11]. In the final, or manifestation, stage of SAR, the plant responds to normally virulent pathogens in a resistant manner [3]. Manifestation of SAR is associated with the expression and activity of a set of SAR genes [17] including the previously described *PR *genes. An earlier, more rapid or more abundant accumulation of these SAR proteins may be the molecular basis for systemic resistance. The physiological function of many of these genes has not been determined but increases in peroxidase activity in induced cucumber [18], chitinase activity in *Arabidopsis *and cucumber [19], as well as antifungal properties *in vitro *[20] suggest that these proteins play a role in producing a resistant state.

Isolation and characterization of *Arabidopsis *mutants has been a powerful approach to decipher the mechanism of SAR. By screening a collection of T-DNA tagged *Arabidopsis *lines for mutants that fail to develop SAR following induction with avirulent *Pseudomonas syringae *pv *tomato *(*Pst*), the *defective *in *induced resistance 1-1 *(*dir1-1*) mutant was identified [21]. The *dir1-1 *mutant was not compromised in basal resistance and, interestingly, overexpression of DIR1 did not enhance disease resistance or lead to a constitutive SAR response. Petiole exudates, enriched for phloem sap, collected from SAR-induced wild-type leaves were effective in inducing the SAR marker gene *PR-1 *when infiltrated into wild-type or *dir1-1 *plants, suggesting that the long-distance SAR signal was present in these wild type petiole exudates and that *dir1-1 *can perceive this signal. However, exudates similarly collected from *dir1-1 *leaves were incapable of inducing *PR-1 *expression in wild-type leaves, suggesting that this mutant is defective either in the synthesis of the SAR mobile signal or its transport to distant leaves [21]. These data and the fact that *DIR1 *encodes a putative lipid transfer protein led to the hypothesis that DIR1 is involved in long distance signaling and may chaperone a lipid signal to distant leaves during SAR [21,13].

Lipid transfer proteins (LTPs) are ubiquitous in plants and are associated with many developmental and stress response processes [22]. The structure of a number of LTPs has been determined revealing that they possess a consensus motif of eight cysteine residues engaged in four disulphide bridges forming a central hydrophobic cavity which can bind long chain fatty acids [22]. Lascombe et al. [23] determined the structure and lipid binding properties of DIR1 expressed in the yeast *Pichia pastoris *using fluorescence and X-ray diffraction. DIR1 shares some structural and lipid binding properties with the LTP2 family. *In vitro*, DIR1 can bind two monoacylated phospholipids and contains two proline-rich SH3 domains. SH3 domains participate in protein-protein interactions in numerous proteins [23]. Lascombe et al. postulate that the DIR1 SH3 domains may play a role in interacting with the putative SAR signal receptor in distant leaves. A number of studies implicate glycerolipids [24,25], methyl salicylate (MeSA) and azelaic acid (AA) as SAR long distance signal candidates [26-28]. Overexpression/SAR studies in *dir1-1 *identified two tobacco DIR1 orthologs indicating that DIR1 is important for SAR in both *Arabidopsis *and tobacco [29]. A recent paper by Chanda et al. [30] provides evidence suggesting that glycerol-3-phosphate (G3P) may also be a SAR long distance signal.

If DIR1 is chaperoning a signal(s) to distant leaves during SAR, we hypothesize that DIR1 accesses sieve elements for long distance movement. Therefore, DIR1 promoter transgenic lines were investigated to localize DIR1 in leaves at the cellular and subcellular levels in healthy untreated plants and during SAR. Our results indicate that the DIR1 promoter directs constitutive expression in seedlings and all leaf cell types. Moreover, although DIR1 expression is reduced upon SAR induction, DIR1 is still expressed in all living cell types comprising the vascular tissue.

## Results

### Localization of DIR1 in leaves during SAR

**Figure 1:**
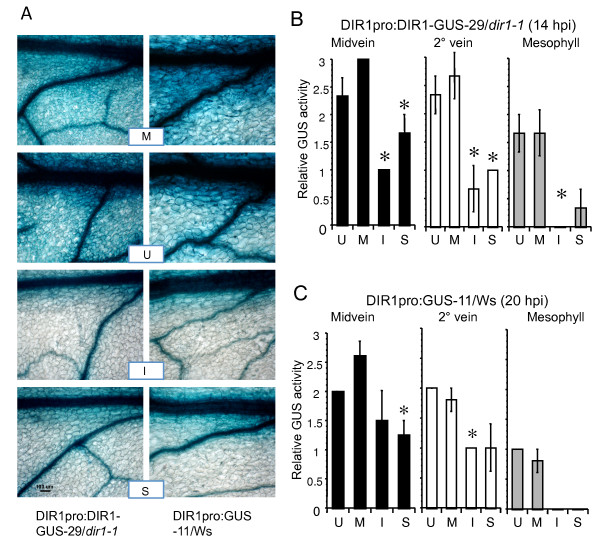
**DIR1 expression in leaves using the DIR1 promoter:GUS plant lines**. **(A) **DIR1pro:DIR1-GUS-29/*dir1-1 *and DIR1pro:GUS-11/Ws plants lines (3.5 wpg) were left untreated (U), mock-inoculated (M) or inoculated with 10^6 ^cfu ml^-1 ^of SAR-inducing *PstavrRpt2 *(I) and harvested at 14 hpi, 20, 40 hpi and subjected to histochemical GUS analysis. Staining pattern were similar at all time points, therefore 14 hpi is shown for DIRpro:DIR1-GUS-29/*dir1-1 *and 20 hpi leaves for DIRpro:GUS-11/Ws. Systemic leaves were also collected from plants that were SAR induced (S). Representative leaves from each line were photographed in a single sitting without adjusting microscope settings and two different leaves are shown. The bar represents 100 μm. Measurement of relative GUS activity in **(B**) DIR1pro:DIR1-GUS-29/*dir1-1 *and **(C) **DIR1pro:GUS/Ws. Leaves from the experiment presented in panel A were scored using a subjective relative scale of 0 to 4, with 0 representing little GUS staining and 4 representing intense GUS staining. U = uninoculated, M = mock-inoculated, I = inoculated leaf from SAR-induced plants, S = systemic leaf from SAR-induced plants. The asterisk (*) denotes a significant difference (student's t test) between mock-inoculated leaves and leaves induced for SAR. This experiment was repeated once with similar results.

Previous RNA and protein gel blot expression studies indicated that DIR1 is expressed constitutively at low levels in rosette leaves of 3 to 4 week old plants and its expression is reduced after SAR induction [21]. If DIR1 is involved in the long distance signaling stage of the SAR pathway, it is possible that DIR1 is expressed in the phloem, specifically companion cells, providing it direct access to the phloem for long distance movement. Moreover, expression limited to the phloem would be consistent with low DIR1 RNA and protein levels observed in whole leaves [21]. DIR1 expression in leaves was examined using the ß-glucuronidase (GUS) reporter gene. The GUS reporter was chosen to amplify the weak DIR1 expression signal and allow visualization of DIR1 expression in various tissues and at the cellular level. Transgenic plant lines were created in which the DIR1 promoter region was placed upstream of GUS in wild-type (ecotype Ws) plants or upstream of a DIR1-GUS fusion in the *dir1-1 *mutant background (see Methods for details). A number of plant lines were examined at four weeks post germination (wpg) for GUS activity before and during SAR. DIR1pro:GUS in Ws lines 1, 11, 23 and DIR1pro:DIR1-GUS in *dir1-1 *lines 3, 15, 29 were mock-inoculated (10 mM MgCl_2_), inoculated with SAR-inducing avirulent *Pst *(*avrRpt2*) or left untreated. Similar results were observed in all plant lines (Figure 1 and Additional Files 1, 2) Inoculated leaves and uninoculated systemic leaves from the same plant were collected at 14 or 20 hours post inoculation (hpi), stained for GUS activity and observed using light microscopy. Under low magnification, abundant GUS activity was observed in untreated and mock-inoculated leaves in the vasculature and mesophyll cells in both the DIR1pro:GUS-11 and DIR1pro:DIR1-GUS-29 lines. In contrast, less intense GUS staining was observed in inoculated and systemic leaves of both transgenic lines (11, 29) inoculated with avirulent *Pst *(Figure 1A). Due to differences in cell density and vacuole size of cells in the midvein, secondary vein and mesophyll, it is not possible to compare GUS activity levels between these tissues. Therefore GUS activity was measured separately in each of these tissues using a relative scale of 0 to 4, where 0 represents little to no GUS activity and 4 represents intense GUS activity or staining (Figure 1B, C) to quantify the observed reduction in GUS activity observed in Figure 1A. Intense staining occurred in the midvein and secondary veins in mock-inoculated or untreated leaves of both the DIR1pro:GUS-11 and DIR1pro:DIR1-GUS-29 lines, whereas the level of GUS activity was reduced in inoculated and uninoculated systemic leaves of plants inoculated with SAR-inducing *Pst *(*avrRpt2*). A similar reduction in GUS activity was observed in mesophyll cells of inoculated or systemic leaves collected from plants induced for SAR compared to untreated or mock-inoculated leaves (Figure 1). Comparable results for DIR1pro:GUS-23 in Ws and DIR1pro:DIR1-GUS-3 in *dir1-1 *are presented as Additional Files 1,2 and 3.

**Figure 2:**
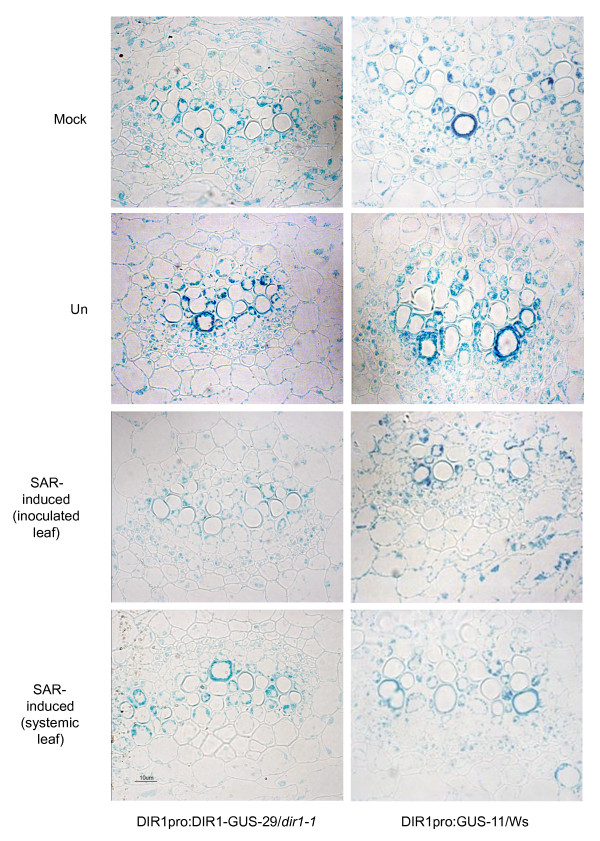
**Cellular localization of DIR1 in leaves**. Leaves from experiments presented in Figure 1 were collected and stained for GUS. Leaves were embedded, sectioned and photographed. Representative sections through untreated (un), mock-inoculated (mock) and SAR-induced (inoculated and systemic) leaf midveins of DIR1pro:DIR1-GUS-29/*dir1-1 *(14 hpi) and DIR1pro:GUS-11/Ws (20 hpi) plants are displayed.

These studies indicate that the *DIR1 *promoter region initiates expression of GUS and DIR1-GUS throughout the leaf and confirms previous RNA gel blot data [21] that DIR1 expression is reduced after SAR induction with *Pst *(*avrRpt2*). DIR1 expression in the vasculature was examined in more detail to determine if DIR1 is expressed in phloem cells using both DIR1pro:DIR1-GUS-29/*dir1-1 *and DIR1pro:GUS-11/Ws lines. GUS-stained leaf and petiole midveins from 4 week-old plants were embedded, sectioned and viewed under high magnification. GUS activity was present in all living cell types including the developing xylem tracheary elements, xylem parenchyma, phloem and phloem parenchyma in midveins of untreated, mock-inoculated, inoculated and systemic leaves from plants induced for SAR (Figure 2). DIR1 expression was reduced, but still detectable in all cell types of the midvein in leaves induced for SAR, including both companion cells and sieve elements of the phloem (Figure 2 and Additional File 4). DIR1-GUS activity was also observed in all cells of untreated petiole midveins (see Additional file 5HI). Therefore, DIR1 is expressed in the phloem before and during SAR induction and may access the phloem for long distance movement during SAR.

Expression of DIR1 in seedlings, roots and flowers was also examined using the DIR1pro-DIR1-GUS-29/*dir1-1 *line. DIR1-GUS activity was observed throughout seven-day old seedlings including the roots, trichomes and in flowers and flower bolts of mature plants (see Additional file 5A-G).

### Reduction in DIR1 expression during SAR induction is *Pst*-dependent

**Figure 3:**
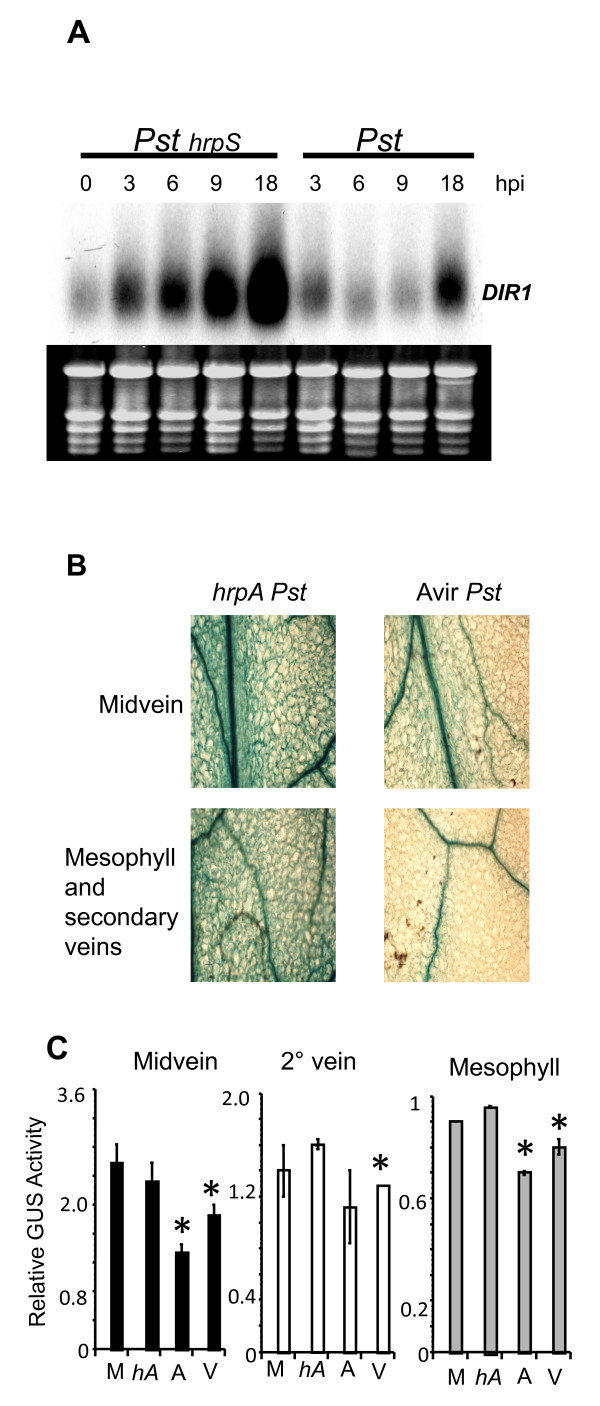
**Reduction in DIR1 expression during SAR is *Pst*-dependent**. **(A) **Plants were vacuum-infiltrated with 10^8 ^cfu ml^-1 ^*hrpS Pst *or *Pst*, followed by RNA gel blot analysis of DIR1 expression at 0,3,6,9 and 18 hpi. Total RNA before blotting is shown to indicate equal RNA loading per well. This experiment was repeated once with similar results. **(B, C) **DIR1pro:DIR1-GUS-29/*dir1-1 *plants were inoculated with 10^6 ^cfu ml^-1 ^*PstavrRpt2 *(Avir) or *hrpA Pst*. Inoculated leaves were collected at 12 hpi and photographed **(B) **and relative GUS activity was determined in midveins, secondary veins and mesophyll cells using the 0-4 subjective GUS scale. The asterisk (*) denotes a significant difference (student's T- test) between mock-inoculated (M) and leaves inoculated with *hrpA *(*hA*) or avirulent (A) or virulent (V) *Pst ***(C)**. This experiment was repeated once with similar results.

A number of studies have demonstrated that virulence effectors delivered by the Type III Secretion System (T3SS) of *Pst *are involved in suppressing *Arabidopsis *cell wall-mediated basal resistance which includes the formation of cell wall callose appositions near *Pst *colonies and the expression of a number of secreted proteins including some LTPs [31-33]. We hypothesized that the reduction in DIR1 expression after inoculation with *Pst *observed in this and our previous study [21] could be the result of T3SS delivery of virulence effectors into the plant cell. To test this hypothesis, DIR1 expression was monitored in wild-type plants inoculated with either virulent *Pst *or a *hrpS Pst *mutant. A high inoculum dose was used (10^8 ^cfu ml^-1^) because nonpathogenic *Pst hrp *mutants do not reliably induce host transcriptional responses at the lower doses [34] typically used in Arabidopsis-*Pst *inoculation experiments. Leaves were collected at 3,6,9 and 18 hpi for RNA gel blot analysis. The T3SS is not functional in *hrpS *mutants and therefore no *Pst*-encoded virulence effectors would be delivered into the plant cell [35,36]. DIR1 was expressed at low levels in untreated leaves and its expression increased from 3 to 18 hpi after infection with *hrpS Pst *(Figure 3A). In leaves inoculated with wild-type virulent *Pst*, DIR1 expression was reduced at 6 and 9 hpi, but this suppression was attenuated by 18 hpi (Figure 3A). These data demonstrate that reduction in DIR1 observed after inoculation with *Pst *is not a response by the plant, but rather a consequence of the delivery of *Pst *virulence effectors into the plant cell.

To examine which cell types are affected by *Pst *virulence effectors, DIR1-GUS expression in the DIR1pro:DIR1-GUS-29/*dir1-1 *line was monitored after inoculation with wild type *Pst *and a *hrpA Pst *mutant that does not make the major pilus protein, HrpA and therefore cannot form the T3SS Hrp pilus or deliver effectors into the plant cell [36]. The *hrpA *mutant or wild-type virulent or avirulent *Pst *(*avrRpt2*) were inoculated (10^6 ^cfu ml^-1 ^dose) into DIR1pro:DIR1-GUS-29/*dir1-1*. Inoculated leaves were collected at 6 and 12 hpi, stained and scored for GUS activity. Similar results were obtained at both 6 and 12 hpi, therefore just the 12 hpi data is presented in Figure 3B and 3C. Mock-inoculated leaves and leaves from plants inoculated with *hrpA Pst *displayed high GUS activity in the midvein, secondary vein and mesophyll cells compared to leaves inoculated with virulent (data not shown) or avirulent *Pst *(Figure 3B). These visual results were corroborated by determining the relative GUS activity using the subjective GUS scale as described above. GUS activity was reduced in the midvein, secondary vein and mesophyll cells in leaves inoculated with either avirulent or virulent *Pst *as compared to leaves inoculated with *hrpA Pst *(Figure 3C). Therefore inoculation with virulent or avirulent *Pst *leads to suppression of DIR1 expression in the midvein, secondary vein and mesophyll cells of leaves in a T3SS-dependent manner.

### DIR1 is targeted to the cell wall

Lipid transfer proteins enter the endoplasmic reticulum (ER) and secretory pathway as preproteins under the direction of a short, N-terminal ER entry peptide of 20 to 26 amino acids that is cleaved after entry into the ER. The mature proteins are secreted outside the cell and are typically associated with cell walls [37-39], although several of these proteins have been discovered intracellularly within protein storage vacuoles or glyoxisomes [40,41]. The functionality of the predicted DIR1 signal sequence was examined by *Agrobacterium*-mediated transient transformation with T-DNA encoding full-length DIR1 fused to the EYFP (enhanced yellow fluorescent protein) reporter (*35S:DIR1-EYFP*), truncated DIR1 lacking the putative signal sequence fused to EYFP (*35S:DIR1^Δ1-25^-EYFP*) or *35S:EYFP *into *Nicotiana tobaccum *followed by laser scanning confocal microscopy to localize EYFP fusion proteins in tobacco leaf epidermal cells.

**Figure 4:**
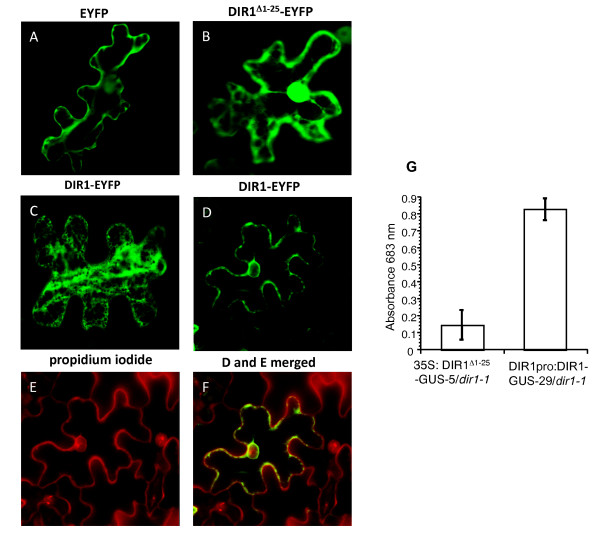
**DIR1 ER signal sequence directs secretion of the protein to the apoplast**. Fusion proteins consisting of full length DIR1 fused to EYFP (DIR1-EYFP) and DIR1 lacking its signal sequence (DIR1^Δ1-25 ^-EYFP) were expressed in *Nicotiana tabaccum *leaves via *Agrobacterium*-mediated transient expression. Fluorescent proteins were visualized in epidermal cells after 48 hours using confocal microscopy. DIR1-EYFP expression exhibited two distinct patterns. **(A) **Fluorescence in the region of the cortical ER and **(D) **the nuclear envelope and cell periphery. Expression of DIR1^Δ1-25^-EYFP and EYFP is shown in **(B) **and **(C)**, respectively. Propidium iodide staining of the plant cell wall is illustrated in **(E)**, and extensive colocalization of DIR1-EYFP with the propidium iodide signal is demonstrated in **(F)**. Subcellular localization experiments were performed three times with similar results. **(G) **IWFs were collected from untreated leaves of 35S:DIR1^Δ1-25^-GUS-5/*dir1-1 *and DIR1pro:DIR1-GUS-29/*dir1-1*. GUS activity was determined by measuring the absorbance at 683 nm. This experiment was repeated 2 additional times with similar results.

Localization of DIR1^Δ1-25^-EYFP was identical to that of the EYFP control, such that fluorescence was observed in 60 of 60 cells at the cell periphery, in cytoplasmic strands and also within the nucleus (Figure 4A,B). Detection of these proteins in the nucleus was likely due to passive diffusion from the cytosol. The 27 kDa EYFP protein, as well as the DIR1^Δ1-25^-EYFP fusion are smaller than the 60 kDa exclusion limit of nuclear pores [42] such that nuclear detection of cytosolic fluorescent fusion proteins is commonly observed in plant cells [43]. DIR1-EYFP exhibited two distinct patterns of localization. In a small number of cells (5/60), DIR1-EYFP was detected in a discrete network particularly enriched near the plasma membrane (Figure 4C) coincident with the cortical ER. In a majority of cells, (55/60), DIR1-EYFP was localized to the nuclear and cell periphery (Figure 4D). Tobacco epidermal cells have a large central vacuole largely restricting the cytoplasm to a thin layer near cell boundaries, making it difficult to distinguish between plasma membrane and cell wall localization. To confirm that DIR1-EYFP was secreted to the cell wall, cells were counterstained with propidium iodide, a dye which accumulates in the apoplast as it is excluded by intact plasma membranes [44,45]. DIR1-EYFP partially colocalized (Figure 4F) with the propidium iodide signal (Figure 4E), demonstrating that the signal sequence directed secretion of DIR1-EYFP out of tobacco epidermal cells into the cell wall. Patches of DIR1-EYFP signal did not colocalize with propidium iodide, but rather with regions surrounding the nucleus and the cell periphery indicating that some DIR1 molecules localize to the ER secretory system and perhaps the cytosol.

**Figure 5:**
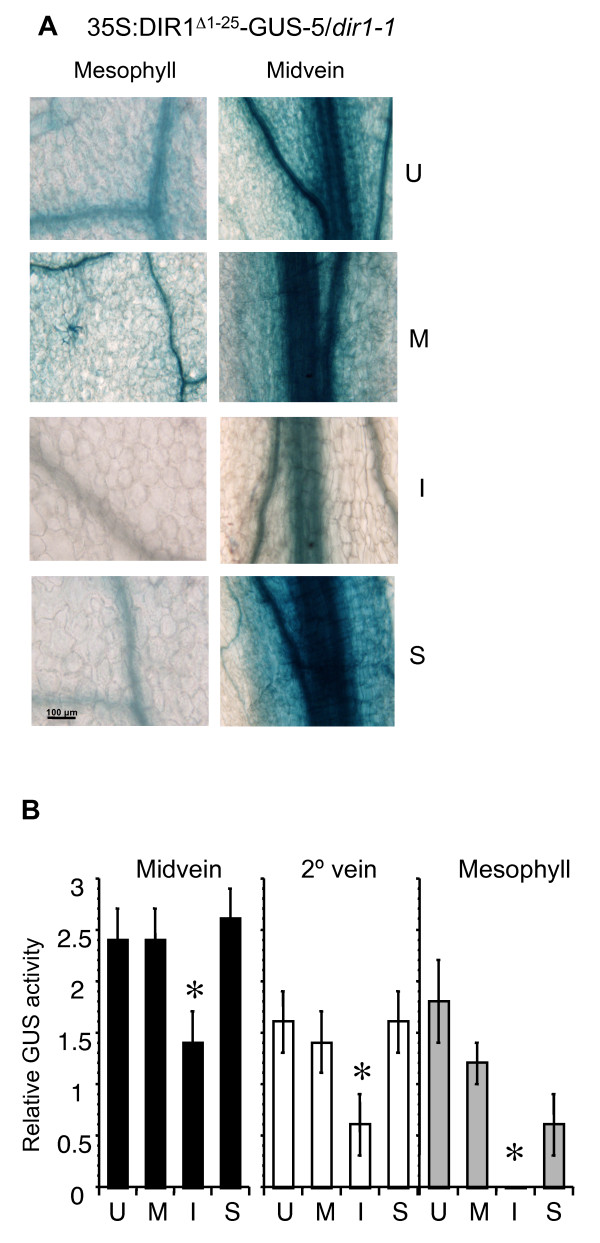
**Localization of DIR1 lacking its signal sequence**. 35S:DIR1^Δ1-25^-GUS-5/*dir1-1 *plants were left untreated (U), mock-inoculated (M) or induced for SAR with *PstavrRpt2 *(10^6 ^cfu ml^-1^). Inoculated (I) and systemic leaves (S) were collected from inoculated plants at 14 hpi. Leaves were stained for GUS activity and photographed in **(A) **or GUS activity levels were determined in the midveins, secondary veins and mesophyll cells of untreated (U), mock-inoculated (M) or leaves induced for SAR (I and S) in **(B)**. The asterisk (*) denotes a significant difference (student's t test) between untreated and inoculated leaves from SAR-induced plants (I). This experiment was repeated once with similar results.

Transgenic lines that express DIR1 lacking its signal sequence in the *dir1-1 *mutant (35S:DIR1^Δ1-25^-GUS in *dir1-1*) were constructed and used to demonstrate the functionality of the DIR1 signal sequence in *Arabidopsis*. A number of lines were characterized (see Methods) and line 5 was chosen for further study. GUS activity in the leaves of 35Spro: DIR1^Δ1-25^-GUS-5/*dir1-1 *line was monitored by inoculating leaves with 10^6 ^cfu ml^-1 ^*Pst (avrRpt2) *followed by GUS staining at 14 hpi. Similar to DIR1 promoter-directed expression (Figures 1 and 2), GUS activity in the 35Spro: DIR1^Δ1-25^-GUS-5/*dir1-1 *line was higher in untreated and mock-inoculated leaves compared to leaves inoculated with avirulent *Pst *(Figure 5A,B and Additional File 6). DIR1^Δ1-25^-GUS was expressed in all cell types of the leaves similar to DIR1 promoter-driven expression of DIR1-GUS. These data indicate that expression from the 35S promoter, like that from the DIR1 promoter region, is reduced in response to inoculation with *Pst*. However, unlike DIR1 promoter-directed expression, 35S promoter-directed expression of DIR1^Δ1-25^-GUS in the midvein and secondary vein of systemic leaves of inoculated plants was similar to untreated or mock-inoculated leaves (Figure 5A, B and Additional File 6). Other researchers have also observed a reduction in 35S promoter-driven expression after pathogen inoculation. For example, expression of GUS in *35S:GUS *transgenic pear was significantly reduced following infection with *Erwinia amylovora *[46] and in *Arabidopsis *and tobacco roots following infection with *Heterodera *and *Globodera *nematodes [47].

To demonstrate that the DIR1 signal sequence does target DIR1 to the cell wall in *Arabidopsis*, intercellular washing fluids (IWFs) were collected from DIR1pro:DIR1-GUS-29/*dir1-1 *and 35S:DIR1^Δ1-25^-GUS-5/*dir1-1 *untreated leaves from 4 week old plants. IWFs consist of cell wall associated proteins and molecules and provide information about the soluble molecules associated with plant cell walls [48,49]. IWFs collected from DIR1pro:DIR1-GUS-29/*dir1-1 *and 35S:DIR1^Δ1-25^-GUS-5/*dir1-1 *leaves were assayed for GUS activity (see Methods). IWFs from DIR1^Δ1-25^-GUS plants displayed low GUS activity while IWFs from DIR1-GUS plants displayed high GUS activity (Figure 4G). Therefore when DIR1 possesses its native signal sequence, DIR1-GUS activity is detected in IWFs which are enriched for soluble cell wall proteins. However, little GUS activity was detected when the native DIR1 signal sequence was removed. These results corroborate the tobacco immunofluorescence analysis demonstrating that the native DIR1 signal sequence targets DIR1 to the cell wall in *Arabidopsis*.

### Expression of DIR1-GUS or DIR1^Δ1-25^-GUS rescues the SAR defect in *dir1-1*

**Figure 6:**
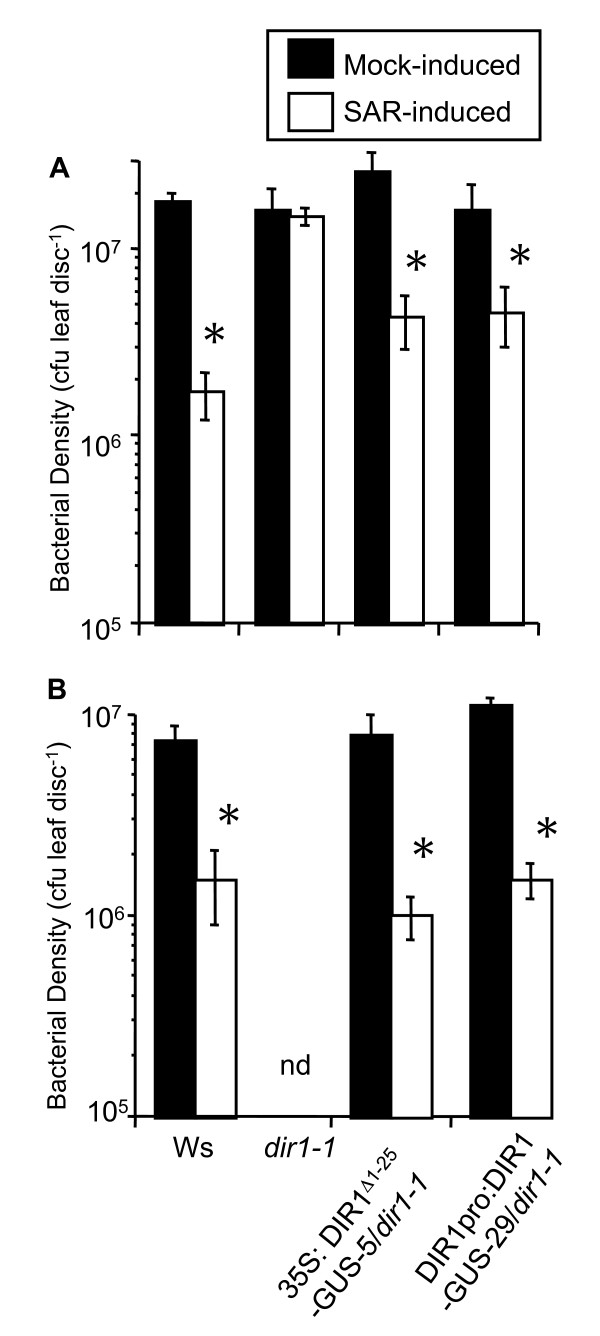
**Expression of DIR1-GUS or DIR1^Δ1-25^-GUS rescues the SAR defect in *dir1-1***. SAR assays were conducted on Ws, *dir1-1*, DIR1pro:DIR1-GUS-29/*dir1-1 *and 35S: DIR1^Δ1-25^-GUS-5/*dir1-1 *by inoculating with 10 mM MgCl_2 _(mock-induced) or inducing for SAR with *Pst-avrRpt2 *(SAR-induced) in 1 to 2 lower leaves, followed by challenge inoculation with virulent *Pst *in distant leaves 2 days later. Bacterial density determination was performed in challenged leaves 3 dpi. Asterisks (*) denote a significant difference (student's t-test) in bacterial densities between challenged distant leaves of mock- and SAR-induced plants. Representative results are presented in **(A) **and **(B) **and these experiments have been repeated numerous times with similar results (see text for details). nd = not determined.

We hypothesize that DIR1 may be involved in long distance signaling during SAR and travel cytoplasmically via the phloem and/or cell to cell. Evidence to date indicates that proteins destined to travel in the phloem in *Arabidopsis *are made in companion cells and enter sieve elements via companion cell-sieve element plasmodesmata [50-52]. However, DIR1 is targeted to the cell wall via the secretory system and according to current cell biology knowledge, DIR1 would have no access to the cytosol and plasmodesmata. We hypothesize that DIR1's targeting signal sequence is cleaved or becomes nonfunctional upon SAR induction allowing it to remain in the cytosol with access to plasmodesmata. If this was true, then DIR1 without its signal sequence may still function during SAR. To test this hypothesis, SAR assays were performed with DIR1pro:DIR1-GUS-29/*dir1-1 *and 35S:DIR1^Δ1-25^-GUS-5/*dir1-1 *lines plus Ws and *dir1-1*. Plants were either induced for SAR with 10^6 ^cfu ml^-1 ^*Pst (avrRpt2) *or mock-inoculated on two lower leaves, followed by challenge inoculation with 10^5 ^cfu ml^-1 ^virulent *Pst *in distant leaves two day later. Bacterial densities were monitored in challenged leaves at 3 dpi. Wild-type Ws plants were SAR-competent as demonstrated by the 10-fold reduction in *Pst *levels in plants induced for SAR versus those that were mock-inoculated, while the *dir1-1 *mutant displayed high levels of *Pst *in plants that were or were not induced for SAR (Figure 6A). Both transgenic lines expressing either DIR1-GUS or DIR1^Δ1-25^-GUS were SAR competent as demonstrated by the 6-fold and 4-fold decrease, respectively, in *Pst *levels in induced versus mock-inoculated plants (Figure 6A). A replicate experiment is shown in Figure 6B in which the transgenic lines displayed a 7- to 8-fold SAR response compared to 5-fold in Ws. Results similar to Figures 6A and 6B were observed using additional transgenic lines (DIR1pro;DIR1-GUS/*dir1-1 *lines 3, 15 and 35S: DIR1^Δ1-25^-GUS/*dir1-1 *lines 17, 20) providing evidence that expression of DIR1-GUS or DIR1^Δ1-25^-GUS restores the SAR defect in the *dir1-1 *mutant. More importantly, these data suggest that removal of the DIR1 signal sequence has no deleterious effect on DIR1's ability to participate in SAR.

## Discussion

The non-specific lipid transfer proteins (LTPs) comprise a large, multigene family present in numerous plant species [39]. LTPs are basic polypeptides of approximately 7-9 kD, whose key structural feature is the LTP fold formed by four disulphide bridges between eight conserved cysteine residues [22]. The LTP fold forms a tunnel-like cavity and *in vitro *studies indicate it accommodates various lipids, including phospholipids, fatty acids, glycolipids, prostaglandin and jasmonic acid [53-58]. Due to their ability to bind lipids *in vitro*, LTPs were originally hypothesized to traffick lipids between intracellular membranes [59]. However, this function seems unlikely as a number of LTPs have been demonstrated to be synthesized as preproteins containing an ER signal sequence such that the mature proteins are secreted to the apoplast [37].

Although the biochemical mechanisms involved are not clear, LTP proteins play important roles in plant defense against pathogens. Several LTPs exhibit antimicrobial activity *in vitro *[60,61] and overexpression of select LTP or LTP-like proteins leads to enhanced local resistance against bacterial and fungal pathogens in *Arabidopsis *and tobacco [62,63]. DIR1 is the first LTP protein whose function in pathogen resistance is defined genetically, as the *dir1-1 Arabidopsis *mutant is impaired in systemic resistance to *Pst *(SAR) but local resistance responses remain intact. Furthermore, 35S promoter-mediated overexpression of DIR1 does not lead to enhanced basal resistance or a more robust SAR response [21], strongly suggesting that DIR1 does not participate directly in defense against *Pst*, but instead plays a role in systemic disease signaling.

The overall goal of this study was to investigate the signaling role of DIR1 during SAR by localizing it at the tissue, cellular and subcellular levels. A number of transgenic lines were created in which the DIR1 promoter region was placed upstream of GUS or a DIR1-GUS fusion in Ws and *dir1-1*, respectively. Examination of these lines indicated that the DIR1 promoter region initiated expression of GUS and DIR1:GUS in seedlings, roots and floral tissues and in all living cells including the veins and mesophyll cells of untreated and mock-inoculated leaves. This was somewhat unexpected as we had hypothesized that DIR1 expression might be limited to the vasculature which would explain the constitutive, but low levels of DIR1 expression observed in leaves [21], while still providing DIR1 access to the phloem for movement during SAR. During SAR induction, DIR1-GUS expression was reduced in mesophyll cells and vascular tissue of inoculated and distant systemic leaves of plants induced with SAR-inducing *Pst *(*avrRpt2*). RNA gel-blots [21] revealed that DIR1 transcript levels declined following SAR induction in leaves. Therefore reduced GUS activity observed in SAR-induced DIR1pro:GUS transgenic plants is the result of decreased transcription driven by the DIR1 promoter region.

Reduction in DIR1 expression could be part of the SAR response or could be due to *Pst*-derived effector molecules delivered into plant cells. To test this hypothesis, expression of DIR1 in leaves inoculated with SAR-inducing avirulent *Pst*, virulent *Pst*, or with a *Pst hrpS *mutant, was determined using RNA gel blot analysis. DIR1 expression was reduced in leaves inoculated with virulent *Pst *at 6 and 9 hpi, however by 18 hpi, DIR1 expression was no longer suppressed. Reduction in DIR1 expression was not observed in leaves inoculated with *Pst hrpS*. Instead, DIR1 transcripts accumulated abundantly at 3, 6, 9 and 18 hpi. A high inoculum dose was used (10^8 ^cfu ml^-1^) because nonpathogenic *Pst hrp *mutants do not reliably induce host transcriptional responses at the lower doses [34] typically used in *Arabidopsis*-*Pst *inoculation experiments. Similar experiments with the DIR1pro:GUS or DIR1pro:DIR1-GUS plant lines using a lower inoculum level (10^6 ^cfu ml^-1^) demonstrated that *Pst *Hrp-dependent suppression of DIR1 expression occurs in the midvein, secondary veins and mesophyll cells at 14 and 20 hpi. Numerous *in planta *bacterial growth studies have demonstrated that the infection process proceeds faster in high compared to low dose experiments [64-68]. Therefore, we speculate that the difference in timing of suppression of DIR1 expression in these two experiments is due to the high versus low inoculum doses used.

Collectively, these data suggest that suppression of DIR1 expression occurs through the action of effector molecules delivered through the *Pst *T3SS. This supports numerous studies in which genes associated with *Arabidopsis *cell wall defense, including a number of LTPs, are suppressed in a *Pst *Hrp-dependent manner [31-33]. Transcriptional mechanisms are involved in the *Pst*-mediated downregulation of DIR1 expression in DIR1pro:DIR1-GUS and 35S:DIR1^Δ1-25^-GUS-5 lines, but it is also possible that post-transcriptional mechanisms or DIR1-GUS instability contribute to the observed expression patterns.

Hrp-dependent suppression of DIR1 occurs in all cell types within inoculated leaves and in distant uninoculated leaves. Recently it was discovered that *Pseudomonas syringae *suppresses plant defenses not only in the infected leaf but also in systemic tissues, rendering the plant more susceptible to subsequent infection, a phenomenon known as Systemic Induced Susceptibility (SIS) [64]. SIS observed after *Pseudomonas *infection of *Arabidopsis *requires the bacterial toxin coronatine [69,70], a structural and functional mimic of the defense hormone jasmonic acid [71]. Interestingly, *Pst hrp *mutants are deficient in the production of coronatine [70]. Reduction of DIR1 expression in systemic tissue may therefore involve the action of widely mobile, bacterially produced molecules such as coronatine.

DIR1 expression in the vasculature was examined in more detail to determine if DIR1 has access to the phloem and therefore the potential for movement to distant leaves, a key characteristic of a SAR long distance signal. Microscopic examination of leaf and petiole cross-sections demonstrated that DIR1-GUS expression was observed in all living cell types including developing xylem, xylem parenchyma, mesophyll, phloem parenchyma and phloem. Mature xylem tracheary elements are dead, and as expected appeared empty with no detectable GUS activity. DIR1 expression was reduced but still detectable after SAR induction in all living cell types including companion cells and phloem sieve elements. Therefore DIR1 is present at the right place (companion cells) and the right time (during SAR induction) to participate in long distance signaling during SAR.

The subcellular localization of DIR1 and the functionality of DIR1's predicted signal sequence were examined by transiently expressing DIR1-EYFP fusion proteins in tobacco epidermal cells followed by visualization using confocal microscopy. As expected, EYFP alone and a fusion construct lacking the predicted ER signal sequence (DIR1^Δ1-25^-EYFP) localized to cytosolic strands and diffused into the nucleus while intact DIR1-EYFP localized to the ER, cell periphery and showed colocalization with propidium iodide, an apoplastic marker. DIR1-GUS activity was detected in intercellular washing fluids from plants expressing wild type DIR1, but GUS activity was greatly reduced in IWFs collected from plants expressing DIR1 lacking the signal sequence, corroborating the DIR1-EYFP tobacco localization experiments. Therefore, the DIR1 signal sequence does direct secretion of DIR1 to the cell wall as has been previously observed for other LTPs [37-39], Similar results were also obtained in a recent paper in which DIR-GFP transiently expressed in *Nicotiana benthamiana *was observed to localize to the ER [30]. It is difficult to distinguish the plasma membrane from the cell wall using light microscopy [38] and this may explain why Chanda *et al. *[30] concluded that DIR1 is not secreted to the cell wall. We chose to examine intercellular washing fluids for the presence of DIR1-GUS for two reasons: to overcome the light-microscopy-associated problem of distinguishing the plasma membrane from the cell wall and to demonstrate that DIR1 is secreted to the cell wall in both tobacco and *Arabidopsis*.

Evidence to date indicates that *Arabidopsis *proteins destined to travel in the phloem are synthesized in companion cells and move into sieve elements through plasmodesmata [50-52]. Patches of intracellular DIR1:EYFP were detected in this study, however it is difficult to distinguish the cytosol from the ER and the secretory system. Nevertheless, these data support the idea that some DIR1 protein is present in the cytosol and therefore gains access to the phloem through the cytosol of companion cells. Alternatively, DIR1 could enter the cytosol if the function of the signal sequence is disrupted during SAR induction. It is also possible that pathogen-induced cell membrane disruption during the HR (SAR induction) may allow cell wall proteins including DIR1 to enter cells. In any case, we hypothesized that cytosolic localization of a pool of DIR1 is required for translocation of the long-distance SAR signal. To address this question, a transgenic line was created in which the signal sequence was deleted from DIR1 (35S:DIR1^Δ1-25^-GUS-5 in *dir1-1 *). We chose to use the 35S promoter in the signal sequence lines and the native DIR1 promoter in the DIR1pro:DIR1-GUS/*dir1-1 *lines because our data indicated that DIR1 expression was very low in wild type plants [21], and we wanted to increase the chance of observing DIR1-GUS in at least one of our lines. In retrospect, this was not necessary as DIR1 expression was observed in the DIR1pro:DIR1-GUS/*dir1-1 *lines, however space and funding constraints made it necessary to work with the 35S: DIR1^Δ1-25^-GUS-5/*dir1-1 *lines. Little DIR1^Δ1-25^-GUS was detected in IWFs collected from 35S:DIR1^Δ1-25^-GUS-5/*dir1-1 *plants. Additionally, removal of the signal sequence restricted expression of a DIR1-EYFP fusion to the cytosol in tobacco cells. Expression of DIR1 without its ER signal sequence rescued the SAR defect in *dir1-1 *to the same extent as the entire protein (DIR1pro:DIR1-GUS/*dir1-1*). These experiments suggest that restricting DIR1 to the cytosol does not impair SAR and supports the idea that cytosolic localization of DIR1 is important during the induction stage of SAR. However, we can not rule out the possibility that higher levels of DIR1^Δ1-25^-GUS produced from the 35S promoter are responsible for the SAR competent phenotype observed in 35S: DIR1^Δ1-25^-GUS-5/*dir1-1 *lines.

## Conclusions

DIR1, like a number of other *Arabidopsis *LTPs, is expressed in seedlings, leaves, roots and flowers [72-74] and contains a signal sequence that directs it to the cell wall. Additionally, DIR1 is upregulated during the basal resistance response to *Pst hrp *mutants in a manner similarly observed in other plant-microbe systems [75]. Our results also confirm previous expression studies that LTPs in *Arabidopsis *are the targets of Hrp-dependent suppression by *Pseudomonas syringae *[31-33]. Although DIR1 expression is suppressed by *Pst*, DIR1 is still detected in companion cells during the SAR induction stage and restriction of DIR1 to the cytosol does not impair SAR, suggesting that DIR1 gains access to sieve elements for transport to distant leaves during SAR. In other words, DIR1 is perfectly situated to participate in long distance signaling during SAR.

## Methods

### Plant growth conditions

*Arabidopsis *seeds from wild-type (ecotype Ws), *dir1-1 *and all transgenic *Arabidopsis *lines were surface sterilized, stratified for 2 days at 4 °C and germinated on solid Murashige and Skoog (MS) medium for 5 to 7 days under continuous light. Seedlings were transferred to soil (Sunshine Mix #1), hydrated with 1 g/L 20-20-20 fertilizer and grown for 3-4 weeks at 22°C, 9 h photoperiod at 150 μE m^-2 ^s^-1 ^light intensity and 65-85% relative humidity.

### Pathogen culture and inoculation

Virulent (containing pVSP1) and avirulent (containing pVSP1 + *avrRpt2*) *Pseudomonas syringae *pv. *tomato *DC3000 strains are previously described [65]. SAR experiments at McMaster were sometimes done using the coronatine mutant *Pseudomonas syringae *pv *maculicola *ES4326 (*Psm*) containing *avrRpt2 *strain [69]. No difference was found in terms of the ability to induce SAR. Bacteria were cultured overnight in King's B medium, diluted to either 10^5 ^or 10^6 ^cfu ml^-1 ^in 10 mM MgCl_2 _and pressure infiltrated into the abaxial side of a leaf using a 1 ml syringe without needle. Quantification of *in planta *bacterial levels was performed by dilution plating essentially as described in [6].

### SAR assays

Plant inoculations were initiated on 3.5 to 4 week old plants (24 to 28 days post germination, dpg). SAR was measured by comparing *in planta *growth of virulent bacteria in plants induced for SAR with *Pst-avrRpt2 *(SAR-induced) with growth in plants inoculated with 10 mM MgCl_2 _(mock-inoculated). Plants were SAR-induced by inoculation of two lower leaves with avirulent *Pst *(10^6 ^cfu ml^-1^) or mock-inoculated, followed by challenge inoculation of distant leaves with 10^5 ^cfu ml^-1 ^virulent *Pst *and *in planta *bacterial level determination 3 dpi. Bacterial density measurements were measured in triplicate for each genotype and treatment and were plotted as the mean ± standard deviation. Pairwise statistical comparisons between SAR-induced plants and the mock-inoculated control were conducted using a Student's T-test at a 0.05 level of significance.

### Collection of intercellular washing fluids

Fully expanded leaves of 3 to 4 week old *Arabidopsis *plants were vacuum infiltrated with sterile distilled water for 30 min, blotted with absorbent paper to dry the leaf surfaces, followed by intercellular washing fluid (IWF) collection from leaves by centrifugation at 1000*g *for 30 min at 4°C [76]. 50 leaves produced approximately 200-300 μl IWF, which is less than previously reported [77] likely because leaves from 3-4 week old plants are smaller than those from 5-7 week old plants. IWFs were sampled immediately for GUS activity.

### Subcellular localization

*Nicotiana tabaccum *was grown under a 9 hour light cycle with 150 μE m^-2 ^s^-1 ^light intensity and ambient humidity. When plants were 5-6 weeks old, overnight cultures of *Agrobacterium tumefaciens *grown in LB medium were resuspended in freshly prepared infiltration buffer (50 mM MES, 2 mM sodium phosphate pH 5.6, 0.1 mM acetosyringone, 13.4 mM sucrose) at an O.D. 600 of 0.1 then pressure infiltrated into the abaxial side of a nearly fully-expanded leaf using a 1 ml syringe without needle. 48 hours later, leaf epidermal cells were imaged on a Zeiss Axiovert laser scanning confocal microscope. In some experiments, a 1 μg/ml solution of propidium iodide in water was pressure infiltrated as described 15 minutes before imaging. Using an argon laser, EYFP and propidium iodide were stimulated at 514 nm and 405 nm respectively, and detected with filter sets at 505-530 nm and 588-614 nm.

### Preparation of tissue, GUS activity and light microscopy

Harvested leaves were washed three times in 50 mM sodium phosphate pH 7.0 and vacuum infiltrated for 30 min with X-glucuronide staining solution consisting of 1 mM X-glucuronide (Rose Scientific, Edmonton), 0.02% Silwet L-77, 20% methanol (v/v), 10 mM EDTA, 40 mM sodium phosphate pH 7.0 [78]. Staining was developed by overnight incubation at room temperature. Stained leaves were fixed for 24-72 h in a solution containing 3.7% formaldehyde in 50 mM sodium phosphate pH 7.0 then dehydrated and cleared by a graded ethanol series. Whole leaves were wet mounted in 70% ethanol and photographed with a Nikon DXM1200F digital camera mounted on a Leica Labrolux 12 microscope using either PHACO2 25/0.5 or Leitz Wetzlar EF 10/0.5 objective lenses. GUS activity or staining intensity was scored on a relative scale of 0 to 4, with 0 representing no visible staining and 4 being extremely intense staining. Tissue to be embedded was excised from the stained and partially dehydrated leaf and further fixed for 2-24 h in a solution containing 1.85% formaldehyde, 5% glacial acetic acid and 63% ethanol by volume. Tissue was then completely dehydrated in a graded ethanol series and embedded in Spurr's resin (Marivac, Inc., Montreal). Spurr's resin blocks were sectioned into 1 μm sections using a Reichert-Jung Ultracut ultramicrotome. Sections were fixed to a glass slide using heat and a portion of each slide was counterstained with an aqueous solution of 0.1% saffranin-o then destained in water. Slides were coverslipped in Permount^® ^(Fisher Scientific, Hampton NH) and photographed using a Zeiss AXIO imager D1 microscope fitted with EC Plan-NEOFLUOR 10/0.3 and 100/1.3 objective lenses.

### Construction of DIR1-GUS transgenic lines

A 1266-bp fragment corresponding to the DIR1 promoter region upstream of the initiation codon was PCR amplified, including engineered restriction sites, from *Arabidopsis *Ws genomic DNA using forward primer 5'-CTTCTGCAGCATTATGGTGTTTTCCTTTG and reverse primer 5'-GTGGATCCTTGTGGTGTTGAAATGAATG. The engineered *Pst*I and *Bam*HI restriction sites were then used to ligate the promoter fragment into the respective sites of pCAMBIA1391Z binary vector (Cambia, Australia) upstream of the GUS reporter gene, generating DIR1pro:GUS construct.

A 1613-bp fragment consisting of the native DIR1 promoter sequence immediately upstream of the start codon and DIR1 coding sequence minus stop codon was PCR amplified, including engineered restriction sites, from Ws genomic DNA using forward primer 5'-CTTCTGCAGCATTATGGTGTTTCCTTTG and reverse primer 5'-AGTGAATTCACAAGTTGGGGCGTTG. The PCR product was digested with *Pst*I &*Eco*RI and ligated in-frame upstream of the GUS gene of pCAMBIA1391Xa (Cambia), thus allowing translational fusion of DIR1/LTP to GUS and the resulting vector construct was designated as DIR1pro:DIR1:GUS.

A 397-bp fragment consisting of a truncated DIR lacking signal sequence DIR1^Δ1-25^, and having an engineered ATG, native stop codon, plus entire 3' UTR was PCR amplified from Ws genomic DNA using forward primer 5'-ATGGCGATAGATCTCTGCGGC and reverse primer 5'-TGTTTGGGCCTTGTGTAGTTTTC. The blunt-end PCR fragment was ligated into the *Sma*I site of pBI121 and its sequence was analyzed to be in correct orientation. The recombinant plasmid was digested with *Pst*I and *Eco*RI to release a 3.4-kb fragment, including CaMV35S promoter, and ligated into the *Ps*tI/*Eco*RI sites of pCAMBIA1391Z, resulting in the vector construct designated as 35S: DIR1^Δ1-25^.

A 1114-bp fragment consisting of 35S promoter and a truncated DIR1^Δ1-25 ^was PCR amplified from the pMNDIR1-ssT15 vector (a recombinant pBI121 harbouring the *DIR1 *gene) using forward primer 5'-AGCGGATAACAATTTCACACAGG and reverse primer 5'-AGTGAATTCACAAGTTGGGGCGTTG. The PCR product was digested with *Hind*III and *Eco*RI and cloned in-frame to the GUS gene of pCAMBIA1391Xa, resulting in the construct 35S: DIR1^Δ1-25^-GUS.

Each plasmid was sequenced prior to transferring it into *Agrobacterium tumefaciens *GV3101 via electroporation. Each of these constructs was introduced into *dir1-1 *mutant plants except for the DIR1 promoter-GUS fusion construct which was introduced into wild-type Ws plants via the *Agrobacterium *mediated floral dip transformation technique [79].

### Characterization of DIR1-GUS transgenic lines

Putative primary independent transformants for each construct were selected in the T1 generation by plating ~1800 seeds (T1) on MS medium containing 15 mg/l hygromycin, with a germination frequency of 1.2-1.6%. After selection for 8-10 days, surviving hygromycin-resistant T1 seedlings were transplanted into soil and checked for the presence of the transgene by PCR using GUS primers 32 GUS^+ ^(5'-GTCTGGTATCAGCGCGAAGT-3') and 33 GUS^- ^(5'-GGCACAGCACATCAAAGAGA-3'). Hygromycin-resistant and GUS-containing seedlings were allowed to self-fertilize in order to obtain T2 seed. At least 5 - 10 independent transformants for each of the four constructs were screened for homozygous transgenic plants in the T2 generation based on segregation of the selectable marker gene. Approximately 80 T2 seeds derived from each independent T1 mother plant (Hyg^+^, GUS^+^) were grown on hygromycin-containing MS medium and lines which showed 100% survival were considered homozygous. At least 10-15 T2 seedlings from the same T1 parents were screened for the presence of the transgene using PCR and primers that were specific for each construct, i.e. GUS primers for the DIRpro:DIR1-GUS, 35Spro: DIR1^Δ1-25^-GUS, and DIR1pro:GUS transgenics, and DIR1 specific primers LTP-SSF1 (5'-ATGGCGATAGATCTCTGCGGC-3') and LTP-SSR4 (5'-TGTTTGGGCCTTGTGTAGTTTTC-3') for the 35Spro:DIR1-SS transgenic.

Histochemical assays of ß-glucuronidase (GUS) activity [78] were also performed on leaf samples from 3 to 5 T2 plants derived from at least 4 independently-transformed homozygous DIR1pro:GUS in Ws lines (L1, 4, 11, 23), DIR1pro:DIR1-GUS in *dir1-1 *lines (L1, 3, 4, 15, 14, 17, 29) and 35Spro: DIR1^Δ1-25^-GUS in *dir1-1 *lines (L1, 5, 7, 9, 17). DIR1pro:GUS in Ws lines (1, 11, 23), DIR1pro:DIR1-GUS in *dir1-1 *lines (1,3,29) and 35Spro:DIR1-ss-GUS in *dir1-1 *lines (1, 5, 7) displayed a similar intense uniform staining pattern and were determined to be homozygous as described above and were used in subsequent experiments.

### Construction of *Agrobacterium *expressing EYFP fusion proteins

Binary transformation vectors expressing C-terminal EYFP fusion proteins under the control of the 35S promoter were generated using Gateway^® ^technology from Invitrogen. Sequence encompassing full-length *DIR1 *but lacking a stop codon was PCR amplified from *Arabidopsis *Ws genomic DNA using forward primer 5'-GGGGACAAGTTTGTACAAAAAAGCAGGCTTAATGGCGAGCAAGAAAGCAGCT and reverse primer 5'-GGGGACCACTTTGTACAAGAAAGCTGGGTTACAAGTTGGGGCGTTGGC *DIR1 *lacking its secretion signal sequence but including an engineered start codon was PCR amplified with forward primer 5'-GGGGACAAGTTTGTACAAAAAAGCAGGCTTAATGGCGATAGATCTCTGCGG and the reverse primer described above. EYFP was PCR amplified from pEYFP-N1 (Clontech) with forward primer 5'-GGGGACAAGTTTGTACAAAAAAGCAGGCTTAATGGTGAGCAAGGGCGAGGA and reverse primer 5'-GGGGACCACTTTGTACAAGAAAGCTGGGTTACTTGTACAGCTCGTCCATGCC. PCR products were recombined into entry vector pDONR221 using a BP recombination reaction according to the manufacturer's instructions. LR recombination reactions were performed according to the manufacturer's instructions to introduce these coding sequences into plant binary transformation vector p35S-NEYFP, which is based on pMDC83 [80]. Resulting plasmids 35S:DIR1-EYFP, 35S: DIR1^Δ1-25^-EYFP and 35S:EYFP were sequenced, mobilized into *Agrobacterium tumefaciens *strain GV3101/PmP90 by electroporation and transformed bacteria were selected on 2YT medium containing rifampicin, gentamycin and spectinomycin.

### RNA gel blot analysis

Total RNA was isolated from *Arabidopsis *leaves using the RNAgents total RNA isolation system (Promega). The RNA concentration was determined by absorbance at 260 nm, and then separated on 2% formaldehyde denaturing agarose gels. The RNA was transferred onto Hybond N+ nylon membranes (Amersham, Piscataway, NJ, USA) using 10X SSC, and UV-crosslinked using a Stratalinker (Stratagene, La Jolla, CA, USA) with the auto-crosslink setting. The *DIR1 *gene was amplified from Col-0 genomic DNA using the following primers: DIR1For 5'-AGCAATCCAATCTGGTTCAC-3' and DIR1Rev 5'-TAACATCCGATATTTAGAATAGGAG-3'. The 491bp *DIR1 *fragment was cloned, reamplified using vector primers and labeled with ^32^P-dCTP using the Stratagene Prime-It II random primer labeling kit. RNA blot hybridization with the *DIR1 *probe was performed using PerfectHyb Plus hybridization buffer (Sigma, St. Louis, MO, USA) following the manufacturer's protocol, and then washed with 0.5 × SSC and exposed to film.

## Authors' contributions

RC conceived of most of the experiments and she and her lab members performed the majority of the experiments presented: MN created the transgenic DIR1-GUS and DIR1^Δ1-25^-GUS *Arabidopsis *lines, AM and KH molecularly characterized these lines, HS performed microscopy, including quantifying relative GUS intensity of these lines. MC contributed significantly to writing the manuscript, conceived of and subcellularly localized DIR1 in tobacco epidermal cells, performed disease resistance assays and constructed the *Agrobacterium *35S:DIR1-EYFP, DIR1^Δ1-25^-EYFP and EYFP lines. PF supported MC's tobacco work. RT and SH performed the RNA gel blots of DIR1 expression in response to *hrpPst*. ND provided plant cell biology expertise. All authors read and approved the final manuscript.

## Supplementary Material

Additional file 1 **Supplementary Figure S1. GUS expression in DIR1pro:GUS-23 leaves**. DIR1pro:GUS-23 was left untreated, mock inoculated or inoculated with 10^6 ^cfu ml^-1 ^of virulent *Pst *or avirulent *Pst avrRpt2 *and harvested for histochemical GUS analysis at 20 hpi. Untreated, mock inoculated, inoculated and systemic leaves were processed and photographed as in Figure 1. Click here for file

Additional file 2 **Supplementary Figure S2. DIR1-GUS expression in DIR1pro:DIR1-GUS-3/*dir1-*1 leaves**. DIR1pro:DIR1-GUS-3 in *dir1-1 *was left untreated, mock inoculated or inoculated with 10^6 ^cfu ml^-1 ^of virulent *Pst *or avirulent *Pst avrRpt2 *and harvested for histochemical GUS analysis at 14 hpi. Untreated, mock inoculated, inoculated and systemic leaves were processed and photographed as in Figure 1. Click here for file

Additional file 3 **Supplementary Figure S3. Relative GUS activity in DIR1pro:DIR1-GUS and DIR1pro:GUS lines**. Untreated, mock inoculated, inoculated and systemic leaves from SAR-induced plants in experiments presented in Figure 1 and Supplementary Figures 1 and 2 were scored using the scale described in Figure 1B. Asterisks denote a significant difference between treatment and mock control. Click here for file

Additional File 4 **Supplementary Figure S4. GUS expression in DIR1pro:GUS-11/*dir1-1 *and DIR1pro:DIR1-GUS-29/*dir1-1 *vasculature**. 3.5 week-old DIR1pro:GUS-11/*dir1-1 *was mock inoculated and DIR1pro:DIR1-GUS-29/*dir1-1 *was inoculated with 10^6 ^cfu ml^-1 ^avirulent *Pst avrRpt2*. Leaves were sampled 20 hpi and sectioned through the midvein. Abbreviations: SE - sieve tube element; CC - companion cell (additional SE/CC pairs are circled); Xi - immature xylem vessel; Xm - mature xylem vessel; Xp - Xylem parenchyma. Click here for file

Additional file 5 **Supplementary Figure S5. Localization of DIR1 at various developmental stages in DIR1 promoter- GUS lines**. Various tissues were stained for GUS and photographed. A. Ws seedling 7 dpg B. DIR1pro:DIR1-GUS-29/*dir1-1 *seedling 7 dpg C. 35S:DIR1^Δ1-25^-GUS-5/*dir1-1 *seedling 7 dpg D. DIR1pro:DIR1-GUS-29/*dir1-1 *flower and E. flower bolt F. DIR1pro:DIR1-GUS-29/*dir1-1 *seedling roots and G. root hairs H. Cross-section of DIR1pro:DIR1-GUS-29/*dir1-1 *untreated petiole. I. Cross-section of 35S: DIR1^Δ1-25^-GUS-5/*dir1-1 *untreated petiole. Click here for file

Additional file 6 **Supplementary Figure S6. GUS expression in 35S:DIR1^Δ1-25^-GUS-17/*dir1-1 *leaves**. 35S:DIR1^Δ1-25^-GUS-17/*dir1-1 *was left untreated, mock inoculated or inoculated with 10^6 ^cfu ml^-1 ^of avirulent *Pst avrRpt2 *and harvested for histochemical GUS analysis at 20 hpi. Midveins and mesophyll cells of untreated, mock inoculated, inoculated and systemic leaves were processed and photographed. Relative GUS staining was scored according to the scale in Figure 1B. Click here for file
