# Supplementary material for: Localization of DIR1 at the tissue, cellular and subcellular levels during Systemic Acquired Resistance in Arabidopsis using DIR1:GUS and DIR1:EGFP reporters
Source: BMC Plant Biol. 2011 Sep 6;11:125. doi: 10.1186/1471-2229-11-125 (PMC3180652; doi:10.1186/1471-2229-11-125)
Supplement: Additional file 1 — Supplementary Figure S1. GUS expression in DIR1pro:GUS-23 leaves. DIR1pro:GUS-23 was left untreated, mock inoculated or inoculated with 106 cfu ml-1 of virulent Pst or avirulent Pst avrRpt2 and harvested for histochemical GUS analysis at 20 hpi. Untreated, mock inoculated, inoculated and systemic leaves were processed and photographed as in Figure 1. [file 1471-2229-11-125-S1.PDF]

DIR1pro:GUS-23 in Ws

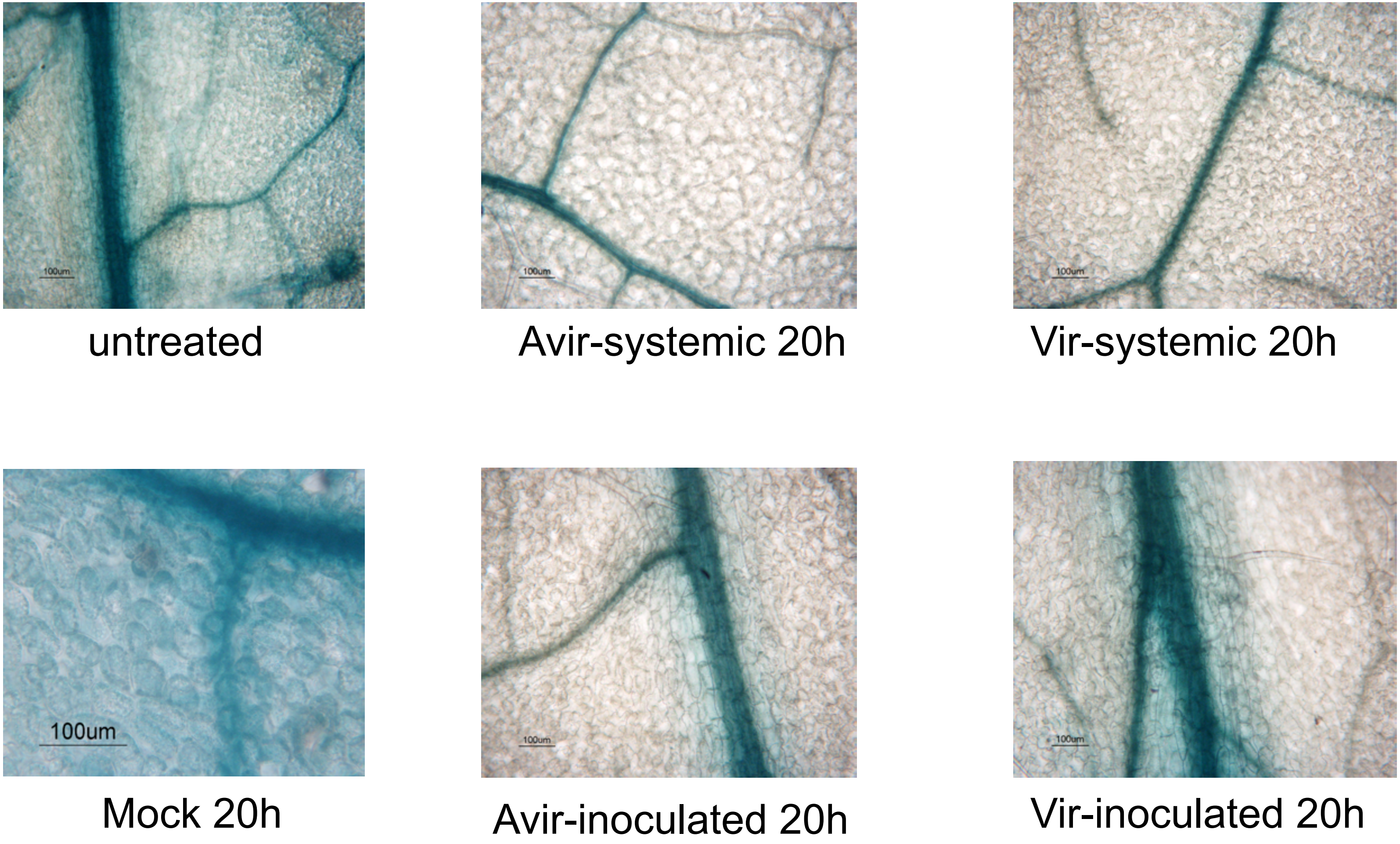

Supplementary Figure S1. GUS expression in DIR1pro:GUS-23 leaves. DIR1pro:GUS-23 was left untreated, mock inoculated or inoculated with 10^6 cfu ml^{-1} of virulent *Pst* or avirulent *Pst avrRpt2* and harvested for histochemical GUS analysis at 20 hpi. Untreated, mock inoculated, inoculated and systemic leaves were processed and photographed as in Figure 1.
