# Supplementary material for: Localization of DIR1 at the tissue, cellular and subcellular levels during Systemic Acquired Resistance in Arabidopsis using DIR1:GUS and DIR1:EGFP reporters
Source: BMC Plant Biol. 2011 Sep 6;11:125. doi: 10.1186/1471-2229-11-125 (PMC3180652; doi:10.1186/1471-2229-11-125)
Supplement: Additional file 3 — Supplementary Figure S3. Relative GUS activity in DIR1pro:DIR1-GUS and DIR1pro:GUS lines. Untreated, mock inoculated, inoculated and systemic leaves from SAR-induced plants in experiments presented in Figure 1 and Supplementary Figures 1 and 2 were scored using the scale described in Figure 1B. Asterisks denote a significant difference between treatment and mock control. [file 1471-2229-11-125-S3.PDF]

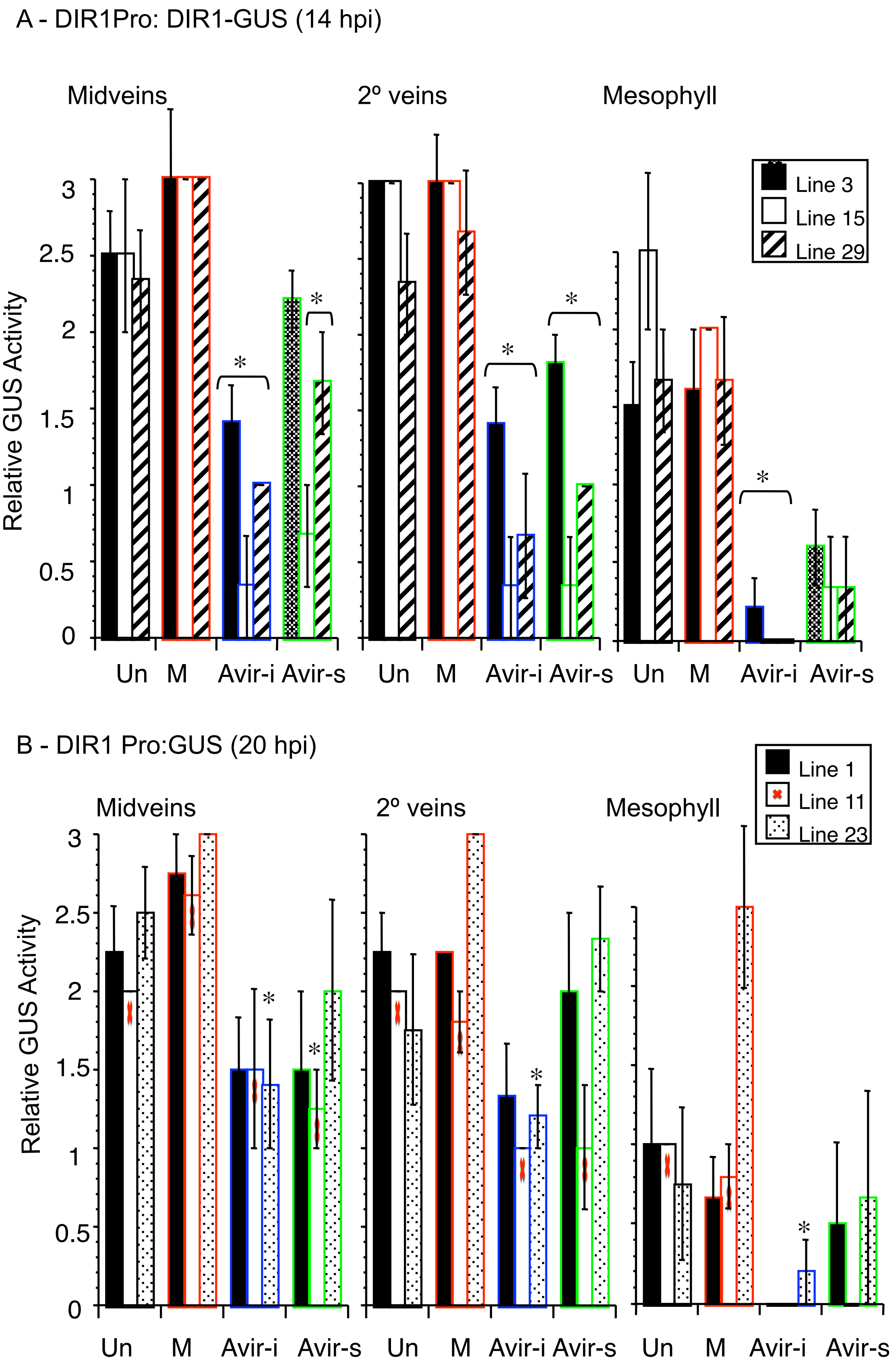

Supplementary Figure S3. Relative GUS activity in DIR1pro:DIR1-GUS and DIR1pro:GUS lines. Untreated, mock inoculated, inoculated and systemic leaves from SAR-induced plants in experiments presented in Figure 1 and Supplementary Figures 1 and 2 were scored using the scale described in Figure 1B. Asterisks denote a significant difference between treatment and mock control.
