# Supplementary material for: Localization of DIR1 at the tissue, cellular and subcellular levels during Systemic Acquired Resistance in Arabidopsis using DIR1:GUS and DIR1:EGFP reporters
Source: BMC Plant Biol. 2011 Sep 6;11:125. doi: 10.1186/1471-2229-11-125 (PMC3180652; doi:10.1186/1471-2229-11-125)
Supplement: Additional File 4 — Supplementary Figure S4. GUS expression in DIR1pro:GUS-11/dir1-1 and DIR1pro:DIR1-GUS-29/dir1-1 vasculature. 3.5 week-old DIR1pro:GUS-11/dir1-1 was mock inoculated and DIR1pro:DIR1-GUS-29/dir1-1 was inoculated with 106 cfu ml-1 avirulent Pst avrRpt2. Leaves were sampled 20 hpi and sectioned through the midvein. Abbreviations: SE - sieve tube element; CC - companion cell (additional SE/CC pairs are circled); Xi - immature xylem vessel; Xm - mature xylem vessel; Xp - Xylem parenchyma. [file 1471-2229-11-125-S4.PDF]

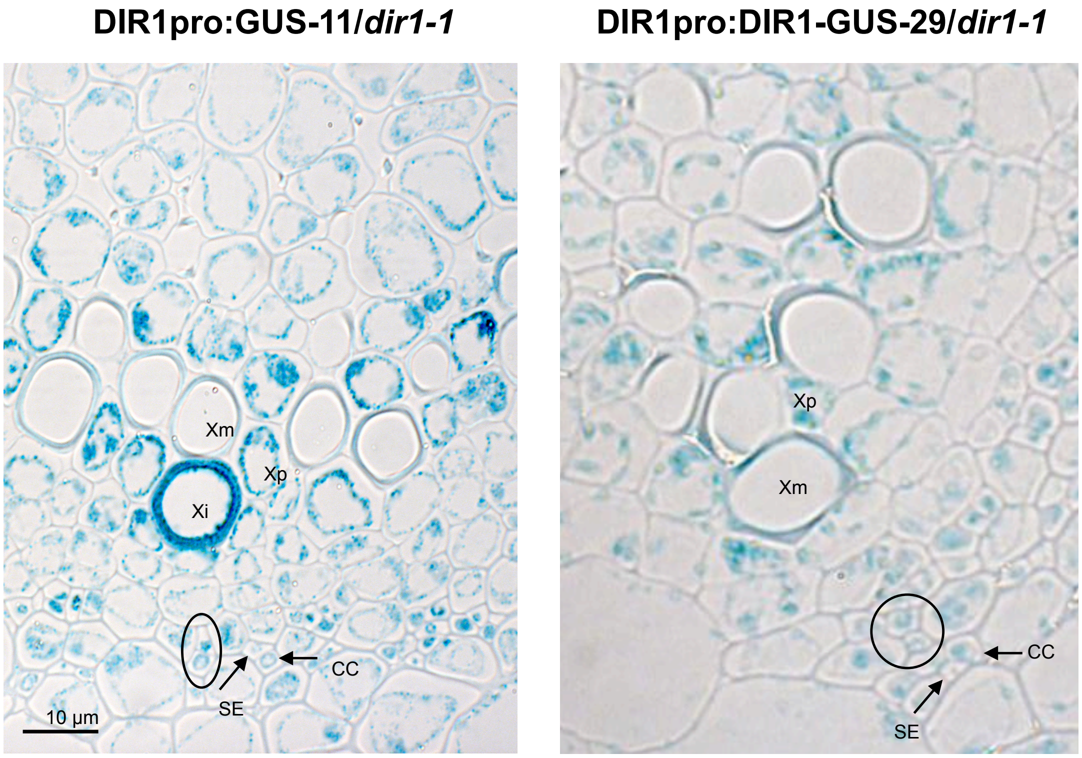

Supplementary Figure S4. GUS expression in DIR1pro:GUS-11*dir1-1* and DIR1pro:DIR1-GUS-29/*dir1-1* vasculature. 3.5 week-old DIR1pro:GUS-11/*dir1-1* was mock inoculated and DIR1pro:DIR1-GUS-29*/dir1-1* was inoculated with 10^6 cfu ml^{-1} avirulent *Pst avrRpt2.* Leaves were sampled 20 hpi and sectioned through the midvein. Abbreviations: SE - sieve tube element; CC – companion cell (additional SE/CC pairs are circled); Xi – immature xylem vessel; Xm – mature xylem vessel; Xp – Xylem parenchyma.
