# Supplementary material for: Localization of DIR1 at the tissue, cellular and subcellular levels during Systemic Acquired Resistance in Arabidopsis using DIR1:GUS and DIR1:EGFP reporters
Source: BMC Plant Biol. 2011 Sep 6;11:125. doi: 10.1186/1471-2229-11-125 (PMC3180652; doi:10.1186/1471-2229-11-125)
Supplement: Additional file 5 — Supplementary Figure S5. Localization of DIR1 at various developmental stages in DIR1 promoter- GUS lines. Various tissues were stained for GUS and photographed. A. Ws seedling 7 dpg B. DIR1pro:DIR1-GUS-29/dir1-1 seedling 7 dpg C. 35S:DIR1Δ1-25-GUS-5/dir1-1 seedling 7 dpg D. DIR1pro:DIR1-GUS-29/dir1-1 flower and E. flower bolt F. DIR1pro:DIR1-GUS-29/dir1-1 seedling roots and G. root hairs H. Cross-section of DIR1pro:DIR1-GUS-29/dir1-1 untreated petiole. I. Cross-section of 35S: DIR1Δ1-25-GUS-5/dir1-1 untreated petiole. [file 1471-2229-11-125-S5.PDF]

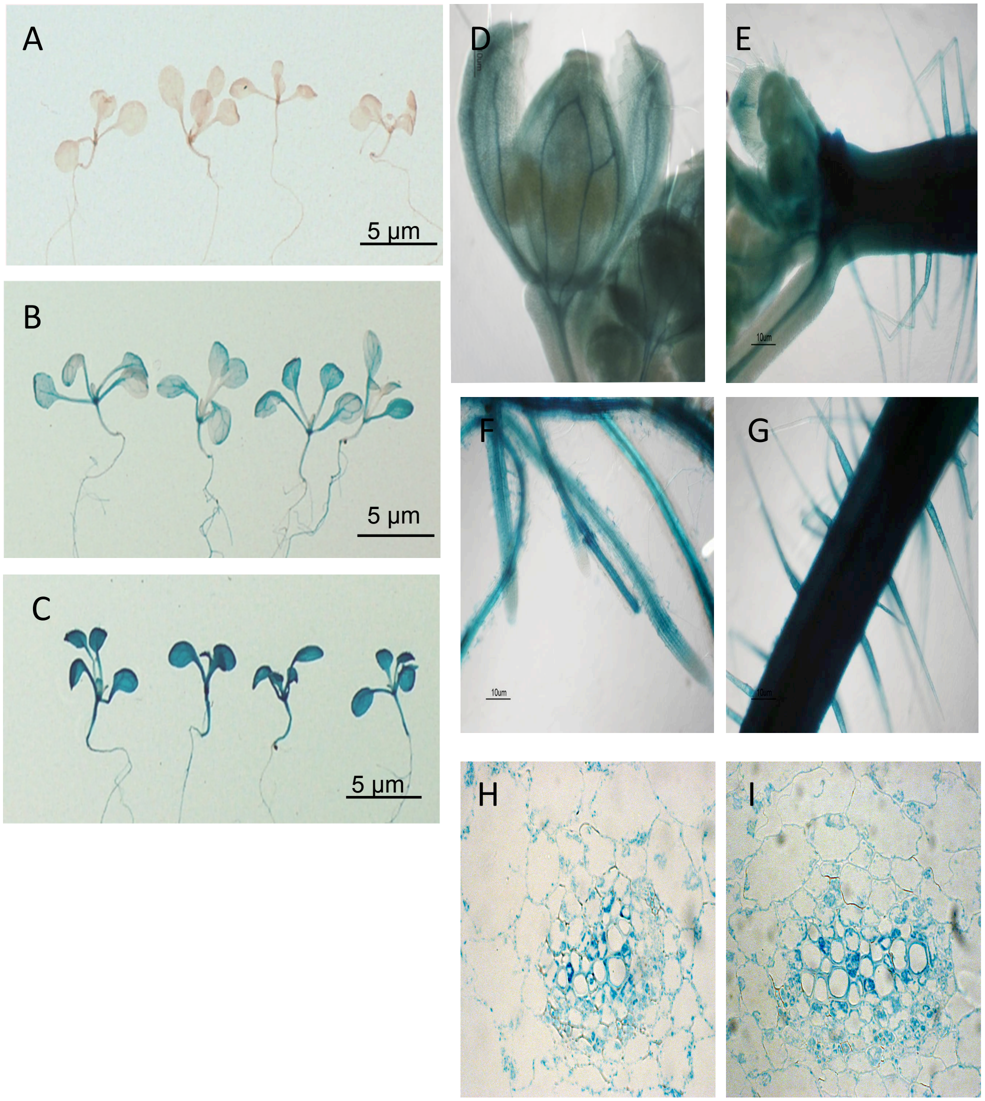

Supplementary Figure S5. Localization of DIR1 at various developmental stages in DIR1 promoter- GUS lines. Various tissues were stained for GUS and photographed. A. Ws seedling 7 dpg B. DIR1pro:DIR1-GUS-29/*dir1-1* seedling 7 dpg C. 35S:DIR1$^{\Delta 1\text{-}25}$-GUS-5/*dir1-1* seedling 7 dpg D. DIR1pro:DIR1-GUS-29/*dir1-1* flower and E. flower bolt F. DIR1pro:DIR1-GUS-29/*dir1-1* seedling roots and G. root hairs H. Cross-section of DIR1pro:DIR1-GUS-29/*dir1-1* untreated petiole. I. Cross-section of 35S: DIR1$^{\Delta 1\text{-}25}$-GUS-5/*dir1-1* untreated petiole.
