# Supplementary material for: Localization of DIR1 at the tissue, cellular and subcellular levels during Systemic Acquired Resistance in Arabidopsis using DIR1:GUS and DIR1:EGFP reporters
Source: BMC Plant Biol. 2011 Sep 6;11:125. doi: 10.1186/1471-2229-11-125 (PMC3180652; doi:10.1186/1471-2229-11-125)
Supplement: Additional file 6 — Supplementary Figure S6. GUS expression in 35S:DIR1Δ1-25-GUS-17/dir1-1 leaves. 35S:DIR1Δ1-25-GUS-17/dir1-1 was left untreated, mock inoculated or inoculated with 106 cfu ml-1 of avirulent Pst avrRpt2 and harvested for histochemical GUS analysis at 20 hpi. Midveins and mesophyll cells of untreated, mock inoculated, inoculated and systemic leaves were processed and photographed. Relative GUS staining was scored according to the scale in Figure 1B. [file 1471-2229-11-125-S6.PDF]

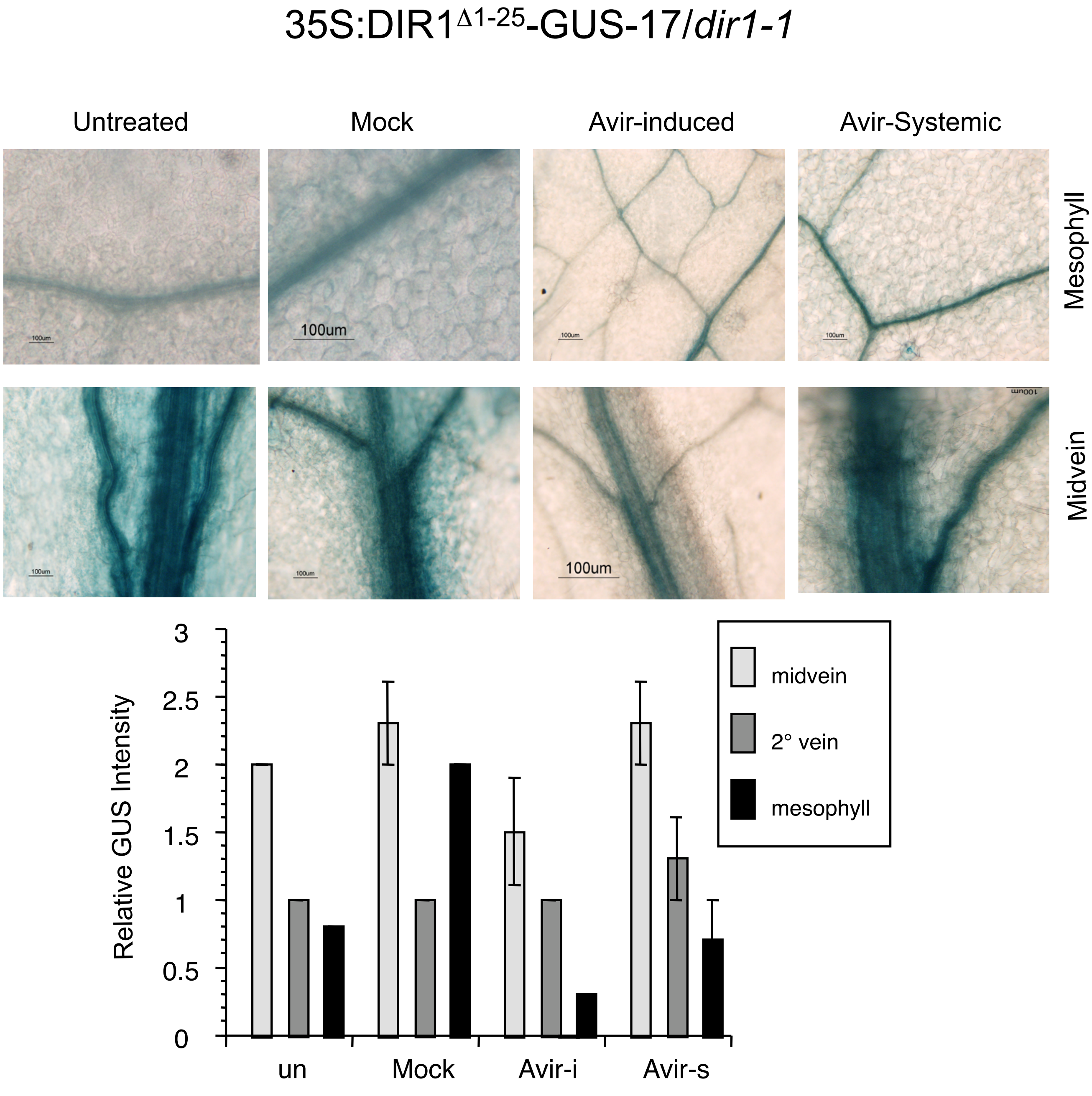

Supplementary Figure S6. GUS expression in 35S:DIR1$^{\Delta 1\text{-}25}$-GUS-17/*dir1-1* leaves. 35S:DIR1$^{\Delta 1\text{-}25}$-GUS-17/*dir1-1* was left untreated, mock inoculated or inoculated with 10^6 cfu ml^{-1} of avirulent *Pst avrRpt2* and harvested for histochemical GUS analysis at 20 hpi. Midveins and mesophyll cells of untreated, mock inoculated, inoculated and systemic leaves were processed and photographed. Relative GUS staining was scored according to the scale in Figure 1B.
